# Who receives abortions at 6- or 16-weeks’ gestation? A case study from Ohio

**DOI:** 10.1080/26410397.2026.2637322

**Published:** 2026-02-25

**Authors:** Pragi Patel, Payal Chakraborty, Bucky Foster, Jacob Kepes, Danielle Bessett, Alison H. Norris, Mikaela H. Smith

**Affiliations:** a Medical Student, College of Medicine, The Ohio State University, Columbus, OH, USA; b Postdoctoral Researcher, Department of Population Medicine, Harvard Pilgrim Health Care Institute and Harvard Medical School, Boston, MA, USA; Department of Epidemiology, Harvard T.H. Chan School of Public Health, Boston, MA, USA; c PhD Student, Department of Epidemiology, Gillings School of Global Public Health, University of North Carolina Chapel Hill, Chapel Hill, NC, USA; d PhD Student, Department of Sociology, College of Arts and Sciences, The Ohio State University, Columbus, OH, USA; e Professor, Department of Sociology, University of Cincinnati, Cincinnati, OH, USA; f Professor, Division of Epidemiology, College of Public Health, The Ohio State University, Columbus, OH, USA; g Research Scientist, Division of Epidemiology, College of Public Health, The Ohio State University, Columbus, OH, USA. *Correspondence*: smith.14017@osu.edu

**Keywords:** abortion, gestation ban, health policy, Ohio, chart review

## Abstract

In the United States, states enacted gestation-based bans following the US Supreme Court case *Dobbs v. Jackson Women's Health Organization*. We examined how patient characteristics differed by gestation at abortion. We used data from a 20% random sample of charts at three Ohio abortion facilities for patients who visited 2014–2018. We used logistic regression to calculate unadjusted and adjusted odds of abortion before 6 weeks and at 16 weeks or after, based on sociodemographic characteristics. We analysed 4,926 charts. Black patients and those of another race had lower odds of abortion before 6 weeks compared to White patients (odds ratio [OR]: 0.30, CI: 0.21–0.42, *p* < 0.001; and OR: 0.64, CI: 0.41–0.995, *p* = 0.048, respectively), as did those from out-of-state (OR: 0.22, CI: 0.11–0. 42, *p* < 0.001). Patients aged 30 and older (compared to those under 25; OR: 1.66, CI: 1.25–2.21, *p* < 0.001), those with a college degree (OR: 2.60, CI: 2.03–3.33, *p* < 0.001), married patients (OR: 1.77, CI: 1.27–2.46, *p* < 0.001), and those without children (OR: 1.29, CI: 1.01–1.66, *p* = 0.041) had higher odds. Patients with a college degree and those without children had lower odds of abortion at 16 weeks or after (OR: 0.67, CI: 0.49–0.91, *p* = 0.010; and OR: 0.75, CI: 0.58–0.97, *p* = 0.030, respectively), while those from out-of-state had higher odds (OR: 2.18, CI: 1.64–2.90, *p* < 0.001). Laws restricting access to abortion create inequitable harms to pregnant people, with particular impacts on patients of colour, those with less education, younger patients, and parents. As states restrict abortion, accessing timely care may become particularly challenging for those experiencing systemic discrimination.

## Plain language summary

Many states have passed laws that ban abortions after a specific number of weeks of pregnancy. In this study, we wanted to learn who might lose access to abortion under these types of bans. To do so, we compared patients who had abortions before six weeks of pregnancy to those who had them at six weeks or after. We also compared patients who had abortions before 16 weeks of pregnancy to those who had them at 16 weeks or after. Our data came from looking through medical charts from three abortion facilities in Ohio for patients who sought care between 2014 and 2018. We found that people who were more likely to have abortions before six weeks were White, older, college-educated, married, not parents, and from Ohio. Similarly, people who were more likely to have abortions before 16 weeks were college-educated, not parents, and from Ohio. States that ban abortion at specific weeks of pregnancy will make accessing timely abortion care more challenging, especially for members of marginalised groups.

## Résumé

Aux États-Unis, après l'arrêt de la Cour suprême dans l'affaire *Dobbs v. Jackson Women's Health Organization*, les États ont adopté des interdictions de l'avortement basées sur l'âge gestationnel. Nous avons examiné comment les caractéristiques des patientes variaient selon l'âge gestationnel au moment de l'avortement. Nous avons utilisé les données d'un échantillon aléatoire de 20% des dossiers de patientes de trois centres d'avortement de l'Ohio pour des consultations entre 2014 et 2018. Nous avons utilisé la régression logistique pour calculer les probabilités ajustées et non ajustées d'avortement avant six semaines et à 16 semaines ou plus, en fonction des caractéristiques sociodémographiques. Nous avons analysé 4926 dossiers. Les patientes noires et celles d'une autre race avaient moins de probabilités d'avorter avant six semaines que les patientes blanches (rapport de cote [RC] : 0,30, IC :0,21-0,42, *p* < 0,001 ; et RC :0,64, IC : 0,41-0,995, *p* =0,048, respectivement), de même que les patientes venant d'un autre État (RC : 0,22, IC : 0,11-0,42, *p* = <0,001). Les probabilités étaient plus élevées chez les patientes âgées de 30 ans et plus (comparativement à celles de moins de 25 ans ; RC : 1,66, IC : 1,25-2,21, *p* < 0,001), les titulaires d'un diplôme universitaire (RC : 2,60, IC : 2,03-3,33, p<0,001), les patientes mariées (RC : 1,77, IC 1,27-2,46, *p* < 0,001) et celles qui n'avaient pas d'enfant (RC : 1,29, IC : 1,01-1,66, *p* = 0,041). Les patientes titulaires d'un diplôme universitaire et celles qui n'avaient pas d'enfant présentaient de plus faibles probabilités d'avorter à 16 semaines ou plus (RC :0,67, IC : 0,49-0,91, *p* = 0,010 ; et RC : 0,75, IC :0,58-0,97, *p* = 0,030, respectivement), alors que les probabilités étaient plus élevées pour les patientes venant d'un autre État (RC : 2,18, IC : 1,64-2,90, *p* < 0,001). Les lois restreignant l'accès à l'avortement engendrent des préjudices inégaux pour les personnes enceintes, touchant particulièrement les patientes de couleur, celles qui sont moins instruites, les plus jeunes et les parents. À mesure que les États limitent l'avortement, l'accès ponctuel à des soins peut devenir particulièrement difficile pour les personnes qui subissent une discrimination systémique.

## Resumen

En Estados Unidos, los estados promulgaron prohibiciones basadas en la gestación después del caso de la Corte Suprema estadounidense *Dobbs c. Jackson Women's Health Organization*. Examinamos cómo las características de pacientes diferían por gestación en el momento del aborto. Utilizamos datos de una muestra aleatoria del 20% de expedientes en tres centros de aborto en Ohio para pacientes atendidas ahí entre 2014 y 2018. Utilizamos la regresión logística para calcular probabilidades no ajustadas y ajustadas de aborto antes de las 6 semanas y a las 16 semanas o más de gestación según las características sociodemográficas. Analizamos 4926 expedientes. Las pacientes negras y aquéllas de otra raza tenían menores probabilidades de aborto antes de las 6 semanas comparadas con las pacientes blancas (razón de momios [RM]: 0.30, IC: 0.21–0.42, *p* < 0.001; y RM: 0.64, IC: 0.41–0.995, *p* = 0.048, respectivamente), al igual que aquéllas que provenían de otros estados (RM: 0.22, IC: 0.11–0.0.42, *p* =<0.001). Las pacientes de 30 años o más (comparadas con aquéllas menores de 25 años; RM: 1.66, IC: 1.25–2.21, *p* < 0.001), aquéllas con título universitario (RM: 2.60, IC: 2.03–3.33, *p* < 0.001), pacientes casadas (RM: 1.77, IC: 1.27–2.46, *p* < 0.001) y aquéllas sin hijos (RM: 1.29, IC: 1.01–1.66, *p* = 0.041) tenían mayores probabilidades. Las pacientes con título universitario y aquéllas sin hijos tenían menores probabilidades de aborto a las 16 semanas o más (RM: 0.67, IC: 0.49–0.91, *p* =0.010; y RM: 0.75, IC: 0.58–0.97, *p* = 0.030, respectivamente), mientras que aquéllas provenientes de otros estados tenían mayores probabilidades (RM: 2.18, IC: 1.64–2.90, *p* < 0.001). Las leyes que restringen el acceso al aborto crean daños inequitativos para las personas embarazadas y afectan en particular a las pacientes de color, a aquéllas con menor nivel educativo, a las pacientes jóvenes y a los padres. A medida que los estados restrinjan el aborto, acceder a servicios oportunos podría ser más difícil para las personas que sufren discriminación sistémica.

## Introduction

In the United States (US), many states have enacted abortion restrictions related to a patient’s pregnancy duration.^1^ Under previous US Supreme Court Rulings, including *Roe v. Wade* (1973), *Planned Parenthood v. Casey* (1992), and *Whole Woman's Health v. Hellerstedt* (2016), states could not ban abortions prior to the third trimester; however, after the US Supreme Court ruling in *Dobbs v. Jackson Women’s Health* (2022), states could pass such restrictions.^2–5^ Legislatures introduced and/or enacted gestation-based restrictions at 6 weeks, 12 weeks, 15 weeks, and 18 weeks.^1^ Prior research has shown that gestation-based bans have led to increases in out-of-state travel,^6^ and that presenting at a later gestational stage can be associated with denial of care due to state limits.^7^ A 2025 summary of the literature on gestation-based abortion bans among nine countries, including the US, finds they can lead to delays in care, forced continuation of pregnancy, opportunity costs (including financial and emotional), extra-legal abortion, self-managed abortion, and challenges receiving referrals to another provider.^8^ Other research on perceptions of gestational stage among abortion patients in one US state shows that almost half of surveyed women underestimated their weeks’ gestation by 5 weeks or more,^9^ which has important implications if patients are faced with gestation-based abortion bans that could make them ineligible for care sooner than anticipated.

Furthermore, research suggests that the impacts of these laws are inequitable.^8^ Data from the Guttmacher Institute from 2008 and 2014 suggests that US abortion patients are disproportionately young, Black, Hispanic, without a college degree, and living with lower income.^10^ In states where Medicaid can be used to cover abortions, the majority of survey respondents in the Guttmacher Institute's 2021–2022 Abortion Patient Survey (62%) did so.^11^ The disproportionate use of abortion by these groups can be understood by looking at structural factors that members of these groups face, including barriers to accessing other forms of healthcare, exposure to medical racism, and living in less-resourced neighbourhoods.^12^ Guttmacher data also shows that in the year leading up to *Dobbs*, abortion-restrictive states had both a higher percentage of Black patients and a higher percentage of patients using financial assistance than abortion-protective states.^13^ Similarly, among a national sample of abortion patients, non-Hispanic Black patients and those with lower income were found to be more likely to have had a procedural abortion, which usually takes place at a later gestation and is also often more costly.^14^ Any bans that limit access to abortion at large will more directly impact those who use abortion in higher proportions, and thus these gestation-based bans particularly harm those who are younger, Black or Hispanic, less educated, or economically disadvantaged.

Similarly, other research from both national and state-specific contexts within the US suggests that Black patients, those with less education, those with lower incomes, younger patients, and patients who live further away from a clinic are more likely to obtain an abortion in the second trimester, while older patients and those with college degrees are more likely to obtain an abortion at or before 6 weeks' gestation.^7,15–20^ Data from a Massachusetts abortion referral programme found that non-White patients had increased odds of experiencing three months or more between pregnancy discovery and programme referral.^9^ Similarly, survey data from Ohio shows that Black patients, those with lower income and education, and younger patients were less likely to discover they were pregnant before 6 weeks,^21^ which may be a contributor to these patients experiencing abortion delays. Importantly, US data based on the 2008 Guttmacher Abortion Patient Survey finds that Black women experience greater delays to care than White women, and that these effects are moderated by income for Black women (but not White women),^22^ highlighting the intersectional nature of these impacts.^23,24^

Patients who are denied care due to being beyond their state’s gestational limit are faced with three primary options: travelling out-of-state for care, self-managing their abortion, or continuing their pregnancy to term. Like the impact of gestation-based bans, members of different social groups experience the implications for these options differently. Travelling for abortion care can be costly,^25–29^ and research from before *Dobbs* suggests that those with less education and lower income, those using Medicaid, and non-Hispanic Black, non-Hispanic Asian, or Hispanic patients were less likely to travel out-of-state for care.^30^ Pre-*Dobbs* estimates predicted that travel distance would increase following the ruling,^31^ and indeed 2023 (post-*Dobbs*) data from the Guttmacher Institute shows that interstate travel has increased.^32^

For those that self-manage their abortion,^33,34^ there have been increasing counts of pregnancy-related criminalisation over the past several decades,^35,36^ and this continues to increase post-*Dobbs*.^37^ In the US, people of colour are more likely to face charges for pregnancy-related crimes than White people.^38,39^ Emergency medicine doctors’ concerns over their ability to provide adequate miscarriage management and abortion care highlight additional burdens for patients should they face these complications from self-managing.^40–44^

For those that continue their pregnancy to term, they face the long- and short-term health risks associated with being pregnant.^45–47^ These risks are exacerbated for pregnancies that are unintended.^48^ At the population level, carrying a pregnancy to term is associated with at least a 14-fold increase in risk of mortality compared to having an abortion.^49,50^ Other research has found that maternal death rates are higher in states with restrictive abortion laws.^51^ Relatedly, research shows higher rates of adverse birth outcomes in states with more restrictive reproductive health policies (including those related to abortion),^52,53^ as well as higher rates of infant mortality in abortion-restrictive states.^54–57^ Other research documents the poorer health and economic outcomes for women who were “turned away” from having their abortion due to being beyond the state’s gestational limit.^58^

In this way, abortion restrictions go against a human and reproductive rights framework that highlights forced pregnancy as a form of discrimination.^8,59–61^ Scholars comparing the US to the international abortion rights landscape emphasise how the *Dobbs* decision goes against global human rights initiatives and makes the US stand in contrast to many other countries that are actively protecting the right to abortion.^62^ Disparities in racial health outcomes are particularly at issue in a US context where rates of adverse maternal and infant outcomes are higher for people of colour.^63^ For example, one study found that pregnancy-related mortality rates for Black women were 3.2 times those for White women, and similarly those for American Indian/Alaskan Native women were 2.3 times those for White women,^64^ a reflection of structural racism and inequity.^65^ Thus, factors associated with pregnancy-related mortality, specifically the ineligibility for abortion due to abortion bans, also carry differential risk by race.

## Ohio as a case study

In this study, we explore the differences in patient characteristics at 6- and 16-weeks’ gestation in Ohio. Ohio has a long history of having a state legislature that is hostile to abortion,^66^ which has passed laws such as a mandatory 24-hour waiting period, mandatory ultrasounds, judicial bypass requirements for minors, a ban on using state funds to pay for abortions, and written transfer agreement requirements.^67^ These types of laws, similar to those passed in other abortion-restrictive states,^68^ are medically unnecessary, create burdens for patients and facilities, and can ultimately lead to clinic closures.^67,69–71^ Post-*Dobbs*, Ohio enacted, for three months, a ban on abortions after detection of embryonic cardiac activity (which usually takes place around six weeks' gestation).^72,73^ Nevertheless, in November 2023, voters in the state passed the Reproductive Freedom Amendment to the state constitution via a ballot initiative.^74^ Since the *Dobbs* decision sent the regulation of abortion back to the states, advocates in several states have sought to protect (or in some cases further restrict) access to abortion care via ballot initiatives.^75,76^ With the success of the ballot initiative in Ohio, advocates have further been able to strike down other restrictions, including the mandatory waiting.^77^

By examining the characteristics of Ohio patients who could be more likely to lose care if they were to be subject to a gestation ban, like those being enacted around the country following the *Dobbs* case*,* we offer insight into the potential inequitable harms of gestation-based bans being currently passed in other states, such as Florida, Iowa, Georgia, and South Carolina.^1^ In particular, given the role that racism plays in limiting access to healthcare for people of colour;^12,23^ the ways that lack of financial resources, combined with parental consent and judicial bypass laws, may limit access to care for young people;^78,79^ and the relationship between gestation bans and out-of-state travel,^6^ we investigate how patients’ state of residence, race, and age differ between these gestational groupings.

Abortion restrictions create delays to care and can push patients beyond a state’s gestational limit. Ohio serves as a useful case study given its legislative history.^67,68^ As a result of the restrictions in the last decade described above, patients have received care at later gestations^67^ and experienced meaningful psychosocial costs in doing so.^80^ Ohio is also demographically similar to much of the Midwest and the US, with a population that is 81% White, 12% Black, and 4% Hispanic, and with 14% living below the poverty level.^81^ Similar to the US, about 1 in 5 Ohioans live in rural areas.^82^ Additionally, Ohio has higher rates of pregnancy-related death than the national average,^83^ and Black people experience almost three times the risk of pregnancy-related mortality than White people (30 deaths per 100,00 births, compared to 12 deaths per 100,000 births),^84^ emphasising the importance of the consequences of gestation-based bans in a state like Ohio.

## Materials and methods

### Data source

We conducted a medical chart review of three standalone abortion facilities in Ohio. We invited all nine standalone abortion facilities providing care in Ohio from 2014 to 2018 to participate. Three abortion facilities agreed to participate in our study. Data represent a 20% annual random sample of abortion patients who had their first visit any time between January 1, 2014 and December 31, 2018. Our study team extracted these data from abortion facility medical charts across 2020–2022. For each facility, we randomly selected charts by first creating a list of all patients who received care during this time period, then assigning them a random number using Excel, and sorting by their assigned random number. The top 20% of patients in this randomly sorted list were included in the extraction sample. Because we used a simple random sample, data are representative of patients who presented at these facilities during this time. The three facilities included in the sample represent approximately 30% of abortions performed in Ohio during the study period and are all in urban locations. During 2014–2018, these clinics provided care up to 13 weeks through 22 weeks of gestation, depending on the clinic and year.

To collect information from patient charts in a standardised manner, our research team developed a data extraction tool that included: patient age, race, marital status, number of children, and state of residence, method of abortion, and gestation at consultation and abortion provision. We developed the tool in conversation with topic experts, abortion providers, and facility staff to ensure validity of the tool and consistent applicability across facilities. The extraction team consisted of a combination of students and staff, and extractors were trained in the extraction process and had completed research ethics training through the CITI programme. This project was approved by the Institutional Review Board (IRB) at The Ohio State University (IRB# 2018H0539) on January 10, 2019. IRB waived requirement for informed consent given that the data are historical medical records, clinics are not in regular contact with historic patients, and contacting the patients would increase their risk of the loss of confidentiality.

### Outcome

We created two separate binary outcomes to measure variation in patient population for those who present for abortion care at earlier and later gestations: gestation before 6 weeks vs. 6 weeks or after; and gestation before 16 weeks or at 16 weeks or after. We calculated weeks' gestation such that 5 weeks, 6 days was coded as before 6 weeks; and 6 weeks, 0 days was coded as 6 weeks or after. Similarly, 15 weeks, 6 days was coded as before 16, and 16 weeks, 0 days was coded as 16 weeks or after. We chose these gestational cut-off points to (1) reflect legislative efforts to ban abortion after 6 weeks or 15 weeks of gestation and (2) balance the distribution of patients included in the sample.

### Patient characteristics

We examined associations between outcomes and the following patient characteristics: race, age, educational attainment, marital status, number of children, in- versus out-of-state, and prior abortion. Referent groups are the largest category. Race was reported as a write-in variable by the patient. We coded patients as being White, Black, or another race (including American Indian, Asian, Pacific Islander, Multiracial, or another race). We chose White as the referent group because it was the largest category, and because it is a socially privileged racial group in the US context. While we collected Hispanic ethnicity data, it was not consistently available across facilities and so we did not include it in our analysis.

We calculated age by subtracting the patient year of birth from the year of first visit and categorised patients as being less than 25, 25–29, and 30 or older. Marital status was reported as never married, married, separated, divorced, widowed, unknown, other, or single. We recoded this to be a binary variable of married versus unmarried. Number of children was recorded as a continuous variable; we recoded it as having no children versus having at least one child. Educational attainment was a 12-category variable that ranged from no schooling to a doctorate degree. We categorised patients as having no college degree (includes no schooling completed, nursery school to 8th grade, some high school, no diploma, high school graduate, diploma or equivalent, some college credit, no degree, and trade/technical/vocational training) or a college degree or higher (includes associate’s degree, bachelor’s degree, master’s degree, professional degree, or doctorate degree).

Patient state was recorded as a write-in based on either patient report or patient’s driver’s license. We recoded patients as being from outside Ohio versus from in-state, and note that state as reported was not necessarily the state from which the patient was travelling for their abortion (e.g. students may have a different permanent residence than their current residence). Finally, we used patient-reported pregnancy history to create a variable measuring whether they had a previous abortion or not. We also examined the method of abortion (medication versus procedural) but do not include it in our regression analyses due to collinearity with gestation at abortion.

### Analysis

To explore the relationships between patient characteristics and timely access to abortion care, we used binary logistic regression to calculate unadjusted odds of having an abortion before 6 weeks (vs. 6 weeks or after) and having an abortion at 16 weeks or after (vs. before 16 weeks). To see if relationships would hold after controlling for potential confounders, we repeated all binary logistic regressions above adjusting for race, age, college, married, at least one child, previous abortion, and year of abortion. We excluded patients from our analysis if they did not receive an abortion or were missing data on any of our evaluated variables. We conducted a sensitivity analysis comparing demographics of the analytic sample to the patients whose charts were excluded for analysis by calculating the proportion of patients in each category with 95% confidence intervals. We conducted all analyses in Stata 16 (StataCorp, College Station, TX).

## Results

### Patient sample

**Figure 1.**
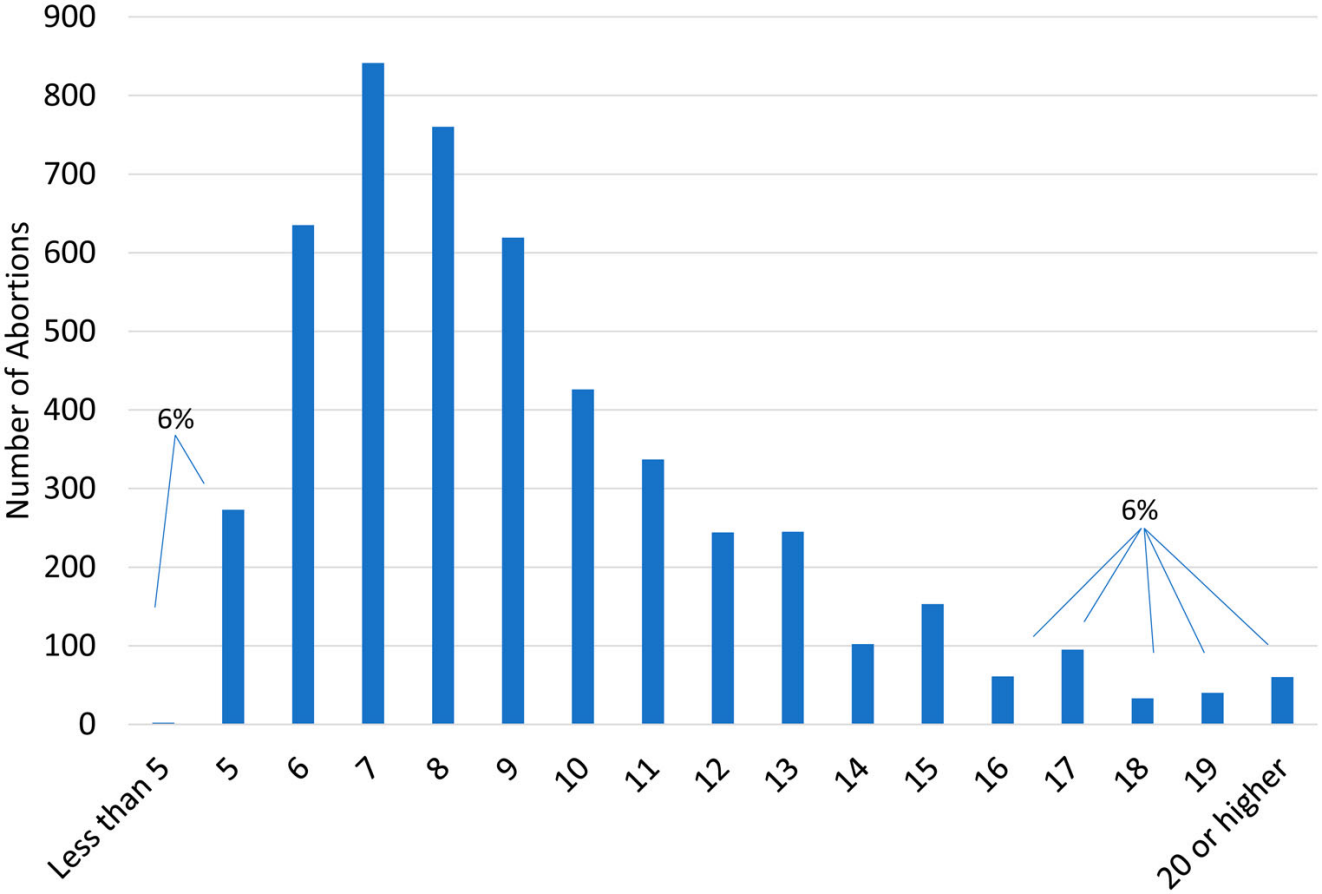
Number of abortions by weeks' gestation among 20% annual random sample of three Ohio abortion facilities, 2014–2018

We sampled 6,349 charts for extraction. We excluded 153 charts from our analysis because they were misfiled and could not be located, and 622 charts for patients who did not have an abortion for the pregnancy that was sampled. We excluded an additional 648 charts with records that were missing key variables. This left us with an analytic sample of 4,926. Overall, our sample had a majority who were White (56%), did not have a college degree (76%), were unmarried (89%), had at least one child (65%), lived in Ohio (87%), and had not previously had an abortion (59%), and a plurality who were under 25 (38%) (Table 1). Six per cent of patients had their abortion before 6 weeks, and another 6% their abortion at 16 weeks or after (Figure 1). Most abortions (86%) were procedural. Compared to the analytic sample (Appendix Table 1), the charts excluded from analysis represented a higher percentage of Black patients (35% vs. 41%), a higher percentage of patients of a race other than Black or White (9% vs. 14%), a higher percentage of patients aged 30 and older (32% vs. 38%), and a higher percentage of married patients (11% vs. 20%). When stratifying by facility (results not shown) we observed that these differences were predominantly driven by a single facility, and so perhaps reflect patient intake processes at that clinic. The missingness patterns by race, age, and marital status thus suggest that data missing in charts may not be at random, and that our findings may under-represent the experiences of people of colour, of older patients, and of married patients.

**Table 1.** Characteristics of sampled patients who received an abortion, 2014–2018 (*N* = 4,926)

	Overall		Less than 6 weeks		6 weeks to 15 weeks, 6 days		16 weeks or greater	
	*N* = 4926		*N* = 275		*N* = 4,362		*N* = 289	
	*N* (%)		*N* (%)		*N* (%)		*N* (%)	
Gestation at abortion								
< 6 weeks	275	(5.6%)						
6–15.6 weeks	4,362	(88.6%)						
≥ 16 weeks	289	(5.9%)						
Race								
Black	1,712	(34.8%)	41	(14.9%)	1,566	(35.9%)	105	(36.3%)
White	2,757	(56.0%)	211	(76.7%)	2,389	(54.8%)	157	(54.3%)
Other	457	(9.3%)	23	(8.4%)	407	(9.3%)	27	(9.3%)
Age								
< 25	1,864	(37.8%)	88	(32.0%)	1,655	(37.9%)	121	(41.9%)
25–29	1,474	(29.9%)	66	(24.0%)	1,332	(30.5%)	76	(26.3%)
≥ 30	1,588	(32.2%)	121	(44.0%)	1,375	(31.5%)	92	(31.8%)
College								
No	3,725	(75.6%)	154	(56.0%)	3,334	(76.4%)	237	(82.0%)
Yes	1,201	(24.4%)	121	(44.0%)	1,028	(23.6%)	52	(18.0%)
Married								
No	4,405	(89.4%)	229	(83.3%)	3,921	(89.9%)	255	(88.2%)
Yes	521	(10.6%)	46	(16.7%)	441	(10.1%)	34	(11.8%)
Has child								
No	1,742	(35.4%)	162	(58.9%)	2,818	(64.6%)	204	(70.6%)
Yes	3,184	(64.6%)	113	(41.1%)	1,544	(35.4%)	85	(29.4%)
State								
In-state	4,285	(87.0%)	266	(96.7%)	3,798	(87.1%)	221	(76.5%)
Out-of-state	641	(13.0%)	9	(3.3%)	564	(12.9%)	68	(23.5%)
Prior abortion								
No	2,890	(58.7%)	172	(62.5%)	2,535	(58.1%)	183	(63.3%)
Yes	2,036	(41.3%)	103	(37.5%)	1,827	(41.9%)	106	(36.7%)
Method								
Medication	691	(14.0%)	127	(46.2%)	564	(12.9%)	0	(0.0%)
Surgical	4,235	(86.0%)	148	(53.8%)	3,798	(87.1%)	289	(100.0%)
Year								
2014	866	(17.6%)	54	(19.6%)	760	(17.4%)	52	(18.0%)
2015	974	(19.8%)	56	(20.4%)	856	(19.6%)	62	(21.5%)
2016	933	(18.9%)	35	(12.7%)	845	(19.4%)	53	(18.3%)
2017	929	(18.9%)	50	(18.2%)	822	(18.8%)	57	(19.7%)
2018	1,224	(24.8%)	80	(29.1%)	1,079	(24.7%)	65	(22.5%)

### Abortions before 6 weeks of gestation

Examining unadjusted odds of having an abortion before 6 weeks vs. 6 weeks or after (Table 2) showed that Black patients were less likely to have an abortion before 6 weeks than White patients (odds ratio [OR]: 0.30, CI: 0.21–0.42, *p* < 0.001) as were those of another race (OR: 0.64, CI: 0.41–0.995, *p* = 0.048). Patients from out-of-state also had lower odds of having an abortion before 6 weeks (OR: 0.22, CI: 0.11–0.42, *P* < 0.001). Patients aged 30 and older had higher odds of having an abortion before 6 weeks, compared to those aged under 25 (OR: 1.66, CI: 1.25–2.21, *p* < 0.001), as did those with a college degree (OR: 2.60, CI: 2.03–3.33, *p* < 0.001), those who were married (OR: 1.77, CI: 1.27–2.46, *p* < 0.001), and those without a child (OR: 1.29, CI: 1.01–1.66, *p* = 0.041).

**Table 2:** Frequency and per cent of patient characteristics for those who had abortions before 6 weeks’ gestation (*N* = 275) or at or after 6 weeks (*N* = 289), and unadjusted and adjusted^a^ odds of having an abortion before 6 weeks’ gestation or at or after 16 weeks, with 95% confidence intervals and *p*-values

	Before six weeks (versus six weeks or after)					
	Unadjusted odds ratio	(95% CI)	*p*-value	Adjusted odds ratio	(95% CI)	*p*-value
Race (ref: White)						
Black	0.30	(0.21, 0.42)	<0.001	0.31	(0.22, 0.44)	<0.001
Other	0.64	(0.41, 0.995)	0.048	0.57	(0.36, 0.89)	0.014
Age (ref: <25)						
25–29	0.95	(0.68, 1.31)	0.739	0.86	(0.60, 1.22)	0.398
≥ 30	1.66	(1.25, 2.21)	<0.001	1.34	(0.93, 1.93)	0.116
College (ref: No)	2.60	(2.03, 3.33)	<0.001	2.13	(1.62, 2.81)	<0.001
Married (ref: No)	1.77	(1.27, 2.46)	0.001	1.11	(0.77, 1.62)	0.568
Has child (ref: Yes)	1.29	(1.01, 1.66)	0.041	1.21	(0.90, 1.63)	0.196
State (ref: In-state)	0.22	(0.11, 0.42)	<0.001	0.17	(0.08, 0.32)	<0.001
Prior abortion (ref: No)	0.84	(0.66, 1.08)	0.179	0.95	(0.72, 1.25)	0.693

	16 weeks or after (versus before 16 weeks)					
	Unadjusted odds ratio	(95% CI)	*p*-value	Adjusted odds ratio	(95% CI)	*p*-value
Race (ref: White)						
Black	1.08	(0.84, 1.40)	0.544	1.22	(0.93, 1.60)	0.161
Other	1.04	(0.68, 1.58)	0.856	1.11	(0.72, 1.70)	0.639
Age (ref: <25)						
25–29	0.78	(0.58, 1.05)	0.105	0.77	(0.56, 1.06)	0.114
≥ 30	0.89	(0.67, 1.17)	0.396	0.85	(0.60, 1.19)	0.337
College (ref: No)	0.67	(0.49, 0.91)	0.010	0.70	(0.50, 0.98)	0.036
Married (ref: No)	1.14	(0.78, 1.64)	0.499	1.22	(0.81, 1.82)	0.345
Has child (ref: Yes)	0.75	(0.58, 0.97)	0.030	0.67	(0.50, 0.90)	0.009
State (ref: In-state)	2.18	(1.64, 2.90)	<0.001	2.31	(1.72, 3.10)	<0.001
Prior abortion (ref: No)	0.81	(0.64, 1.04)	0.098	0.77	(0.59, 1.01)	0.060

^a^Adjustment set includes race, age, college, married, at least one child, and previous abortion.

The adjusted odds of having an abortion before 6 weeks for Black patients and those of another race were lower than those of White patients (aOR: 0.31, CI: 0.22–0.44, *P* < 0.001; and aOR: 0.57, CI: 0.36–0.89, *p* = 0.014, respectively). Those with a college degree had higher adjusted odds of having an abortion before 6 weeks (aOR: 2.13, CI: 1.62–2.81, *p* < 0.001), and those from outside Ohio had lower odds (aOR: 0.17, CI: 0.08–0.32, *p* < 0.001). The odds of having an abortion before 6 weeks were no longer statistically significantly different by age, marital status, or number of children after adjustment.

### Abortions at 16 weeks’ gestation or after

When examining unadjusted odds of having an abortion at 16 weeks or after (Table 2), we found that patients who had a college degree and those without children had lower odds of having their abortion at 16 weeks or after (OR: 0.67, CI: 0.49–0.91, *p* = 0.010; and OR: 0.75, CI: 0.58–0.97, *p* = 0.030, respectively). Those from out-of-state had higher odds of abortion at 16 weeks or after (OR: 2.18, CI: 1.64–2.90, *p* < 0.001). All three remained statistically significant after adjustment. Adjusted odds of having an abortion at 16 weeks or after were lower for college graduates (aOR: 0.70, CI: 0.50–0.98, *p* = 0.036) and those without children (aOR: 0.67, CI: 0.50–0.90, *p* = 0.009). Adjusted odds of having an abortion at 16 weeks or after were higher for out-of-state patients (aOR: 2.31, CI: 1.72–3.10, *p* < 0.001).

## Discussion

Our analysis of patient data from three standalone abortion facilities in Ohio showed that about 6% of patients received their abortions before 6 weeks. Additionally, we found that those who were White, older, college-educated, married, without children, or from Ohio were more likely to have their abortion before 6 weeks. Similarly, about 6% of patients received their abortion at 16 weeks' gestation or after, with those with children, without a college degree, and from out-of-state having higher odds doing so.

CDC data suggest that 40% of patients nationally had abortions before 7 weeks (the lowest gestational cut-off presented) in 2018, and in Ohio 24% of abortions were before 7 weeks.^85^ Our very different finding, that only 6% of patients in the sample had abortions before 6 weeks, may in part be a reflection of the different categories of gestational breakdown available in national data. However, our data also shows that 18% of patients had abortions before 7 weeks. CDC data also suggest that nationally about 4% of abortions took place at or after 16 weeks’ gestation, and 6.5% did so in Ohio, which is similar to our sample. The lower percentage of abortions before 6 or 7 weeks, compared to national trends and state-reported data, may be a reflection of the gestational limits of the facilities included in our study. During the study period, there were facilities in Ohio that provided medication abortion only that were not part of our study; these are represented in the CDC data, and their inclusion increases the overall percentage of abortions at earlier gestational stages for the state as a whole, compared to our sample.

Our findings about who accesses very early abortion care and care later in pregnancy are consistent with literature about the impacts of racism on reproductive healthcare access. Factors such as medical racism and medical mistrust among people of colour can result in delays or decreased access to care,^12,23,86^ and, as in our study, other researchers have found that Black patients have lower odds of having an abortion at or before 6 weeks.^7,15–20,87^ In a survey, Black Ohio patients were also less likely to discover they were pregnant before 6 weeks,^21^ which may be a contributor to these patients being less likely to have an abortion before 6 weeks. While previously published studies also suggest that Black patients were more likely to receive an abortion in the second trimester compared to White patients,^15,17,18^ we did not have the same finding.

Similar to some of our results, other studies examining patient characteristics in the US, and at a single clinic in Illinois, have found that younger patients were more likely to have abortions at 13 weeks or later than before 13 weeks.^17–19,87^ These may reflect differences in patients’ abilities to recognise bodily changes associated with pregnancy, including an increased likelihood of irregular menstrual cycles amongst younger people.^18^ We posit that in addition to delays for younger people driven by late pregnancy recognition and fewer financial resources, patients who are under 18 may experience delays in accessing abortion due to Ohio’s laws requiring parental consent or a judicial bypass for minors to receive abortion care.^67^

While we could not extract data on patient income, our findings related to education may reflect broader socioeconomic status or income. In the US, abortions are often not covered by insurance,^88,89^ and many states have bans on using state funds to pay for abortions.^90^ Thus, having a higher education and/or income may make it easier for patients to receive abortion care earlier in their pregnancy. Cost has consistently been documented as a reason that abortion-seekers experience delays to care.^18,88,91^ Even for individuals who have insurance coverage for abortion, this is not always sufficient for overcoming financial barriers to care.^88,89,92–94^ The real-world impact of these high costs is consequential: among a sample of abortion seekers in three abortion-supportive states, researchers found that almost half experienced a catastrophic health expenditure (CHE; an expenditure that prevents someone from being able to meet their everyday needs);^25^ similarly, another study using abortion cost data and census data concluded that in the majority of states, the out-of-pocket cost of an abortion would qualify as a CHE for median-income earners.^95^ In the US, local abortion funds play an important part in the abortion care ecosystem by providing financial and practical support to people seeking care, and their continued existence may support more timely access to care.^96,97^ Similarly, innovations in abortion provision such as abortion pills via telemedicine will help increase access to those whose economic circumstances prevent them from being able to access in-person care.^98^

Our findings on abortion use and parental status are consistent with national trends, with 60% of patients seeking abortions in the US having at least one child.^85^ It is also possible that the responsibilities of caring for children contribute to delays faced by patients with children, given that a commonly provided reason for delays to abortion includes challenges with arranging and managing appointments and this could be more difficult for those also managing childcare.^18^ Although the majority of people seeking abortions nationally are unmarried,^85^ limited evidence exists regarding patterns related to marital status and gestation at time of abortion.

Our finding that people from Ohio were able to access abortion care earlier in their pregnancy than those travelling from out-of-state is not surprising, given other research showing that patients who travelled further away received abortions at later gestations,^7,15^ and the degree to which geographic proximity may allow patients to schedule an abortion appointment more quickly. Our finding that having had an abortion in the past was not associated with gestation at current abortion suggests that prior experience with abortion is not a meaningful predictor of obtaining care earlier or later in pregnancy.

Recent legislation both in Ohio and nationally has attempted, or succeeded, in limiting abortions at around 6 weeks or 15 weeks of gestation.^99^ Our study reveals that those who would be most likely to lose care under these bans, i.e. younger people, people with children, those with less education, and Black people, are those already experiencing barriers to care and compounded harms in the medical system.^23,100,101^ Multiple factors can combine to contribute to delays in receiving abortions including financial limitations, legislative hurdles, clinic and provider availability, and travel and related logistical issues.^28,102–106^

Furthermore, gestational bans or other barriers can have important, inequitably distributed consequences for those seeking care.^8^ People may be forced to travel outside of their home state for care, but the resources needed to do so (e.g. money for travel and overnight stays, the ability to take time off work or find childcare) may not be available to all.^25–30^ Others may be forced to carry their pregnancy to term, which is associated with increased health risks, including maternal mortality, particularly for Black people and those whose pregnancies are unintended.^45–51,63–65^ Being turned away from abortion care due to being over a state’s gestational limit is also associated with having poorer health and economic outcomes.^58^ Those who choose to have an abortion outside the formal medical system may face higher risk of pregnancy-related criminalisation, particularly people of colour.^35–39^

Human rights and justice frameworks recognise that denying pregnant people access to abortion is a form of discrimination,^59,60^ particularly given the implications for racial and economic disparities as a result of lack of access to holistic sexual and reproductive healthcare.^61,62^ Furthermore, scholars highlight the degree to which the *Dobbs* decision is at odds with global initiatives to protect abortion as a human right.^62^ While *Dobbs* directly targets the right to abortion, it also indirectly threatens access to contraception or the right to marry someone of the same sex, thereby further limiting the right to self-determination especially among pregnancy-capable people.^62^ Even pre-*Dobbs*, a 2018 statement from several professional organisations that provide sexual and reproductive healthcare to pregnancy-capable people highlights how abortion-restrictive laws intertwine to undermine patients’ autonomy and freedom to make their own reproductive decisions.^107^ This lack of access to reproductive choice stands in direct contrast to the tenets of reproductive justice, which are “the human right to maintain personal bodily autonomy, have children, not have children, and parent the children we have in safe and sustainable communities.”^108^ For these reasons, policymakers and clinicians should situate expansive access to abortion care as an essential component of human rights justice.

As states continue to enact gestation-based abortion restrictions, these findings contribute to our understanding of how the impacts of abortion bans will be experienced most by those already marginalised by the healthcare system. We acknowledge important questions remain regarding the work that abortion funds, abortion providers, and other abortion advocacy groups are undertaking in order to help patients overcome barriers to care, such as providing patient navigators, and funding support, and working to pass laws that protect access to abortion at a range of gestational durations, such as Ohio’s reproductive freedom amendment ballot initiative. Our study of Ohio sheds light on a specific geographic context, and future work could look at the implications of changes in access in nearby states, taking a more holistic view of the abortion provision landscape.

### Strengths and limitations

These findings illuminate key characteristics associated with the timing of abortion care without some biases inherent in other research about abortion utilisation, such as low participation rates in surveys conducted with abortion patients, and underreporting of abortion by participants who are sampled from the general population.^109^ Our sample is limited, however, to three independent abortion clinics in Ohio, whose caseloads represent approximately 30% of abortions in the state. Thus, while our sampling structure ensures results are representative of abortions at these sites, they are not necessarily representative of all abortions in Ohio. Additionally, our data were collected from patients who presented at these facilities from 2014 to 2018 and do not include the experiences of patients who sought an abortion but never went to a clinic. Barriers preventing patients from receiving care earlier in their pregnancy are likely similar to those that would prevent someone from scheduling and attending an appointment at an abortion facility, and thus our findings are likely an underestimate of the true impact of barriers on access.

Our dataset is also limited in its sample size. While we found differences between Black and White patients’ gestation at abortion, small sample sizes, when evaluating multiple characteristics at once, prevented us from being able to, for example, explore the interaction of race with other variables such as age, education, or having a child. Additionally, our dataset does not include other variables such as ethnicity, sexuality, gender identity, or income due to a lack of data in the patient charts. Our smaller sample size, along with the relatively low number of abortions before 6 weeks or at or after 16 weeks, may also limit the generalisability of our findings. Caution should especially be taken in the interpretation of results from variables that were not evenly balanced, such as marital status, state of residence, and racial identity of another race besides Black or White. Future research with larger samples of people of colour, or of patients who are young or have less education, could better investigate how barriers to care related to structural racism are intertwined with barriers related to socioeconomic status or age.

## Conclusion

Restrictions that limit access to abortion, including gestational bans, present inequitable harms to people seeking necessary reproductive healthcare. Public opinion research shows that Americans are generally supportive of abortions at 6 and 12 weeks’ gestation,^110^ indicating a disconnect between public attitudes and policy that curtails abortion access. At the time of writing, abortion up to 22 weeks' gestation remains protected in Ohio. This is due in large part to the passage of the reproductive freedom constitutional amendment in November 2023, which represented the first time that a ballot initiative to protect access passed in a state with an abortion-hostile legislature.^74,76^ The success of Ohio’s amendment offers a framework for how access to care at later gestations might be protected in other states (such as Iowa, Florida, South Carolina, and Georgia) that currently have, or are considering, six-week bans. Thus our findings offer an important example of how myriad abortion restrictions, combined with barriers to care rooted in systemic racism, sexism, and classism, limit the ability of people with marginalised identities (e.g. people of colour, young people, and those with fewer economic privileges) from obtaining abortion care very early, or earlier, in pregnancy. In order to counteract these structural barriers to care, we recommend that states expand protections for abortion care by removing gestational limits^8^ and allowing state funds to cover abortion costs.^11^

## Author contributions

*Conceptualisation: PP, AHN, MHS. Data curation: PP, PC, BF, MHS. Analysis: PP, PC, BF, JK, MHS. Funding acquisition: DB, AHN. Investigation: PP, PC, Methodology: PC, MHS. Project administration: PC, MHS. Supervision: PC, DB, AHN, MHS. Visualisation: MHS. Writing: PP, PC, BF, DB, AHN, MHS. Reviewing: PP, PC, BF, JK, DB, AHN, MHS.*

## Supplementary Material

Appendix Table 1
