# Supplementary material for: Who receives abortions at 6- or 16-weeks’ gestation? A case study from Ohio
Source: Sex Reprod Health Matters. 2026 Feb 25;33(1):2637322. doi: 10.1080/26410397.2026.2637322 (PMC13011096; doi:10.1080/26410397.2026.2637322)
Supplement: Appendix Table 1 [file ZRHM_A_2637322_SM0346.docx]

**Appendix Table 1**: Comparison of proportion of patients in the analytic sample versus in charts excluded from analysis with 95% confidence intervals; bold indicates statistically significant difference

|  | Analytic sample (N=4926) | | | Excluded sample (N=637) | | |
| --- | --- | --- | --- | --- | --- | --- |
|  | Proportion | 95% CI lower | 95% CI upper | Proportion | 95% CI lower | 95% CI upper |
| Gestation at abortion |  |  |  |  |  |  |
| < 6 weeks | 5.6% | 4.9% | 6.2% | 5.2% | 3.5% | 7.0% |
| 6-15.6 weeks | 88.6% | 87.7% | 89.4% | 89.8% | 87.5% | 92.2% |
| ≥ 16 weeks | 5.9% | 5.2% | 6.5% | 4.9% | 3.2% | 6.6% |
| Race |  |  |  |  |  |  |
| **Black** | **34.8%** | **33.4%** | **36.1%** | **41.0%** | **36.5%** | **45.5%** |
| **White** | **56.0%** | **54.6%** | **57.4%** | **45.3%** | **40.7%** | **49.8%** |
| **Other** | **9.3%** | **8.5%** | **10.1%** | **13.7%** | **10.6%** | **16.9%** |
| Age |  |  |  |  |  |  |
| < 25 | 37.8% | 36.5% | 39.2% | 34.6% | 30.9% | 38.4% |
| 25-29 | 29.9% | 28.6% | 31.2% | 27.1% | 23.6% | 30.5% |
| **≥ 30** | **32.2%** | **30.9%** | **33.5%** | **38.3%** | **34.5%** | **42.1%** |
| College |  |  |  |  |  |  |
| No | 75.6% | 74.4% | 76.8% | 77.1% | 71.6% | 82.6% |
| Yes | 24.4% | 23.2% | 25.6% | 22.9% | 17.4% | 28.4% |
| Married |  |  |  |  |  |  |
| **No** | **89.4%** | **88.6%** | **90.3%** | **80.9%** | **76.9%** | **84.8%** |
| **Yes** | **10.6%** | **9.7%** | **11.4%** | **19.1%** | **15.2%** | **23.1%** |
| Has child |  |  |  |  |  |  |
| No | 35.4% | 34.0% | 36.7% | 33.5% | 29.8% | 37.2% |
| Yes | 64.6% | 63.3% | 66.0% | 66.5% | 62.8% | 70.2% |
| State |  |  |  |  |  |  |
| In-state | 87.0% | 86.0% | 87.9% | 83.7% | 80.8% | 86.6% |
| Out-of-state | 13.0% | 12.1% | 14.0% | 16.3% | 13.4% | 19.2% |
| Prior abortion |  |  |  |  |  |  |
| No | 58.7% | 57.3% | 60.0% | 60.0% | 56.1% | 63.8% |
| Yes | 41.3% | 40.0% | 42.7% | 40.0% | 36.2% | 43.9% |
| Method |  |  |  |  |  |  |
| Medication | 14.0% | 13.1% | 15.0% | 16.0% | 13.2% | 18.9% |
| Surgical | 86.0% | 85.0% | 86.9% | 84.0% | 81.1% | 86.8% |
